# Lean Body Mass, Interleukin 18, and Metabolic Syndrome in Apparently Healthy Chinese

**DOI:** 10.1371/journal.pone.0018104

**Published:** 2011-03-18

**Authors:** Liang Sun, Frank B. Hu, Zhijie Yu, Huaixing Li, Huaiyu Liu, Xiangdong Wang, Danxia Yu, Hongyu Wu, Geng Zhang, Geng Zong, Yong Liu, Xu Lin

**Affiliations:** 1 Key Laboratory of Nutrition and Metabolism, Institute for Nutritional Sciences, Shanghai Institutes for Biological Sciences, Chinese Academy of Sciences and Graduate School of the Chinese Academy of Sciences, Shanghai, China; 2 Departments of Nutrition and Epidemiology, Harvard School of Public Health, Boston, Massachusetts, United States of America; 3 SIBS-Novo Nordisk Translational Research Centre for PreDiabetes, Shanghai Institutes for Biological Sciences, Chinese Academy of Sciences, Shanghai, China; 4 Shanghai Luwan Center for Disease Control and Prevention, Shanghai, China; 5 Shanghai Zhabei Center for Disease Control and Prevention, Shanghai, China; German Diabetes Centre, University of Duesseldorf, Germany

## Abstract

**Objective:**

We aimed to investigate how lean body mass is related to circulating Interleukin 18 (IL-18) and its association with metabolic syndrome (MetS) among apparently healthy Chinese.

**Methods:**

A population-based sample of 1059 Chinese men and women aged 35–54 years was used to measure plasma IL-18, glucose, insulin, lipid profile, inflammatory markers and high-molecular-weight (HMW)-adiponectin. Fat mass index (FMI) and lean mass index (LMI) were measured by dual-energy X-ray absorptiometry. MetS was defined by the updated National Cholesterol Education Program Adult Treatment Panel III criteria for Asian-Americans.

**Results:**

Circulating IL-18 was positively correlated with LMI after adjustment for FMI (correlation coefficient = 0.11, *P*<0.001). The association with the MetS (odds ratio 3.43, 95% confidence interval 2.01–5.85) was substantially higher in the highest than the lowest quartile of IL-18 after multiple adjustments including body mass index. In the stratified multivariable regression analyses, the positive association between IL-18 and MetS was independent of tertiles of FMI, inflammatory markers and HMW-adiponectin, but significantly interacted with tertile of LMI (*P* for interaction = 0.010).

**Conclusion:**

Elevated plasma IL-18 was associated with higher MetS prevalence in apparently healthy Chinese, independent of traditional risk factors, FMI, inflammatory markers and HMW-adiponectin. More studies are needed to clarify the role of lean mass in IL-18 secretion and its associated cardio-metabolic disorders.

## Introduction

Growing evidence suggests a pivotal role of chronic subclinical inflammation in the pathophysiology of cardio-metabolic disorders [1]. As an active endocrine organ, human adipose tissue secretes multiple pro-inflammatory cytokines, including interleukin 6 (IL-6), tumor necrosis factor (TNF)-α, monocyte chemoattractant protein-1 (2), and IL-18 (3).

Discovered in 1989 as an interferon-γ–inducing factor (4), IL-18 is now recognized as a pleiotropic cytokine related with both innate and acquired immune responses (5). Synergistically with IL-12, IL-18 could induce interferon-γ production from T, B and natural killer cells (6). It also plays a crucial role in pro-inflammatory cascade by stimulating the production of TNF-α and IL-6 (4,7). Elevated IL-18 concentration provided additional information over C-reactive protein (CRP), IL-6 and fibrinogen in predicting cardiovascular mortality for patients with coronary artery disease (8) or incident of coronary events among healthy men (9). Exiting evidence also indicated that circulating IL-18 was positively associated with insulin resistance, metabolic syndrome (MetS) and type 2 diabetes (10–13), suggesting potential applications of IL-18 as an intervention target or prognostic biomarker. However, most studies to date were conducted in western populations and little is known about the effect of IL-18 on cardio-metabolic risk in Asians like Chinese who might have different metabolic susceptibility (14).

Although adipose tissue is believed as one of important sources for IL-18 (3), effects of different body compartments on this pro-inflammatory marker remain controversial. For instance, body mass index (BMI), waist circumference, waist-to-hip ratio, body fat mass and/or fat percentage (fat mass/total body weight) were correlated with levels of IL-18 in some (11,13,15,16), but not in other studies (9,10,12,17). Meanwhile, weight loss following lifestyle changes significantly reduced circulating IL-18 (18,19). Interestingly, one study from 144 healthy men reported that fat-free mass rather than fat mass was positively associated with serum IL-18 (10). Moreover, TNF-α infusion in men enhanced *IL18* mRNA expression in muscles, but not in adipose tissues (20), implicating potential involvement of muscle mass in generating IL-18. However, few studies evaluated specific effects of different body compartments, distinguished by fat mass and lean mass, on relationship between circulating IL-18 and cardio-metabolic disorders. Therefore, we aimed firstly to investigate the relationships between different body compartments with plasma IL-18 and also its association with the MetS; secondly to evaluate to what extent the association was mediated by body-size adjusted lean mass or fat mass, as well as adipokine and inflammation in apparently healthy Chinese men and women.

## Methods

### Ethics Statement

The study was approved by the Institutional Review Board of the Institute for Nutritional Sciences and written informed consent was obtained from all participants.

### Study population

The design and recruitment of the population-based case (BMI≥24.0 kg/m^2^) and control (18≤BMI<24.0 kg/m^2^) study were described in detail elsewhere [21]. Briefly, a total of 1059 (559 overweight/obese and 500 normal-weight) eligible participants aged 35–54 years were recruited from Shanghai, China. Information on demographic variables, health status and behaviors was obtained using a standardized questionnaire [21]. Following a home interview, all participants were asked to fast overnight before a physical examination. Body weight, height, waist circumference and blood pressure were measured using a standardized protocol [21]. Whole-body densitometry was conducted under a Hologic DXA (QDR-4500, Hologic, Waltham, MA, USA; software version 11.2.1).

Individuals without data of dual-energy X-ray absorptiometry (DXA) scan or IL-18 (100, 9.4%), or presumably having acute inflammation (CRP>10 mg/l, 18, 1.7%) were further excluded and final analyses included 941 participants.

### Laboratory methods

Fasting peripheral venous EDTA blood samples were collected and centrifuged at 4°C. All plasma samples were stored at −80°C until analyses. The measurements of total cholesterol, high-density lipoprotein (HDL) cholesterol, low-density lipoprotein (LDL) cholesterol, triglycerides, glucose, glycohaemoglobin (HbA1c), insulin, CRP, lipopolysaccharide binding protein (LBP), IL-6 and high-molecular-weight (HMW)-adiponectin were described previously [21]. The insulin resistance index (homeostatic model assessment of insulin resistance [HOMA-IR]) was calculated according to updated homeostasis model assessment methods (http://www.dtu.ox.ac.uk/).

Plasma IL-18 was analyzed by an ELISA kit from Medical Biological Laboratories (Naku-ku, Nagoya, Japan). The assay has a sensitivity of 12.5 pg/ml with a measurable concentration range of 25.6–1000 pg/ml. The intra-assay coefficients of variation (CV) were 9.9%, 10.8% and 5.0% at 69.7, 345.5 and 2765.5 pg/ml, respectively; while the inter-assay CVs were 6.3%, 5.2% and 10.1% at 160.1, 615.1 and 2621.1 pg/ml, respectively.

### Definition of Metabolic Syndrome

MetS was defined based upon the updated National Cholesterol Education Program Adult Treatment Panel III criteria for Asian-Americans [22] as presenting at least 3 of the following components: 1) Waist circumferences ≥90 cm in men or ≥80 cm in women; 2) Triglycerides ≥1.7 mmol/l; 3) HDL cholesterol <1.03 mmol/l for men or <1.30 mmol/l for women; 4) Blood pressure ≥130/85 mmHg, or current use of anti-hypertensive medications; and 5) Fasting plasma glucose ≥5.6 mmol/l.

Modified MetS was defined as having 2 or more components of MetS without central obesity.

### Calculation of fat mass index and lean mass index

Using fat mass, fat-free mass and bone mass measured by DXA, fat mass index (FMI), and lean mass index (LMI) were calculated as follows and were normalized for body size [23]:

FMI = fat mass/height^2^;
LMI = lean mass/height^2^ = (fat-free mass−bone mineral content) /height^2^.

### Statistical analyses

Log-transformations were performed for IL-18, triglycerides, insulin, HOMA-IR, CRP, IL-6, LBP, and HMW-adiponectin to approximate normality. Analysis of covariance for continuous variables and logistic regression models for categorical variables were applied for the comparison across IL-18 quartiles. Partial Spearman correlation coefficients between IL-18 and body composition, metabolic features and cytokines were calculated by adjustment for age, sex and BMI (not for body composition data). The correlations between IL-18 and FMI or LMI were further adjusted for LMI or FMI (mutually adjusted for each other).

Multivariate logistic regression models were applied to estimate the odds ratios (ORs) for MetS. Adjusted potential confounders included age, sex, lifestyle factors, education level, family history of chronic diseases and BMI, as well as CRP, IL-6, LBP, and HMW-adiponectin. In sex-stratified models, menopause status and hormone use were further controlled in women. The ORs for MetS according to joint classification of IL-18 and FMI, LMI and cytokines were also calculated. Data management and statistical analyses were performed using Stata 9.2 (Stata, College Station, TX). Statistical tests were two-sided and *P* value<0.05 was considered statistically significant.

## Results

### Baseline characteristics

Overweight/obese participants had significantly higher plasma IL-18 levels than their normal-weight counterparts (geometric mean 240.7 (95% confidence interval 232.8–248.8) pg/ml vs. 208.1 (200.7–215.9) pg/ml, respectively; *P*<0.001). Subjects with higher IL-18 concentrations were more likely to be males and alcohol drinkers, and had higher prevalence of MetS, elevated levels of BMI, waist circumference, blood pressure, glucose, HbA1c, insulin, HOMA-IR and triglycerides, and lower concentrations of HDL cholesterol (all *P*<0.05, **Table 1**). They also showed higher CRP, IL-6 and LBP, but lower HMW-adiponectin levels (all *P*<0.01, **Table 1**).

**Table 1:** Characteristics of participants across IL-18 quartile^a^.

	IL-18 quartile				
	Q1 (n = 235)	Q2 (n = 235)	Q3 (n = 236)	Q4 (n = 235)	*P* value
IL-18 (pg/ml)^b^	136.9 (133.4–140.5)	199.0 (196.8–201.3)	257.0 (254.8–259.2)	363.3 (352.9–373.9)	—
Age (yrs)^c^	45.5 (5.4)	46.1 (5.4)	46.6 (5.2)	46.6 (5.3)	0.072
Men (n, %)^d^	36 (15.3)	78 (33.2)	101 (42.8)	130 (55.3)	<0.001
Low physical activity (n, %)	123 (52.3)	115 (48.9)	110 (46.6)	121 (51.5)	0.593
Education levels (n, %)					0.171
0∼9 yrs	66 (28.1)	61 (26.0)	54 (22.9)	69 (29.4)	
10∼12 yrs	119 (50.6)	129 (54.9)	129 (54.7)	127 (54.0)	
>12 yrs	50 (21.3)	45 (19.2)	53 (22.5)	39 (16.6)	
Current smoker (yes, n, %)	27 (11.5)	53 (22.6)	65 (27.5)	83 (35.3)	0.962
Alcohol drinker (yes, n, %)	74 (31.5)	85 (36.2)	79 (33.5)	99 (42.1)	0.039
Family history of chronic diseases (n, %)	101 (43.0)	87 (37.0)	93 (39.4)	99 (42.1)	0.507
Metabolic syndrome (n, %)	56 (23.8)	91 (38.7)	106 (44.9)	137 (58.3)	<0.001
BMI (kg/m^2^)	23.4 (3.7)	24.5 (3.9)	25.3 (4.4)	25.3 (3.9)	<0.001
Waist circumference (cm)	80.1 (10.1)	84.2 (10.5)	86.7 (11.6)	88.1 (11.1)	<0.001
Systolic blood pressure (mmHg)	119.1 (15.6)	124.2 (17.1)	126.4 (17.7)	128.4 (17.1)	<0.001
Diastolic blood pressure (mmHg)	75.1 (10.5)	78.9 (11.3)	80.8 (11.8)	82.2 (11.3)	<0.001
Glucose (mmol/l)	5.79 (0.85)	6.12 (1.53)	6.10 (1.08)	6.41 (1.81)	<0.001
HbA1c (%)	5.62 (0.51)	5.68 (0.84)	5.66 (0.60)	5.85 (0.84)	0.008
Insulin (µU/ml)^b^	7.88 (7.35–8.45)	9.03 (8.43–9.68)	9.62 (8.99–10.29)	10.02 (9.35–10.74)	<0.001
HOMA-IR^b^	0.91 (0.85–0.98)	1.06 (0.99–1.13)	1.12 (1.05–1.20)	1.18 (1.10–1.27)	<0.001
Total cholesterol (mmol/l)	5.26 (1.14)	5.33 (1.19)	5.35 (1.10)	5.27 (1.23)	0.856
LDL cholesterol (mmol/l)	3.20 (0.94)	3.36 (1.02)	3.36 (0.93)	3.34 (1.01)	0.501
HDL cholesterol (mmol/l)	1.56 (0.44)	1.41 (0.41)	1.37 (0.38)	1.24 (0.38)	<0.001
Triglycerides (mmol/l)^b^	1.04 (0.97–1.12)	1.22 (1.13–1.32)	1.39 (1.29–1.49)	1.56 (1.43–1.69)	<0.001
CRP (mg/l)^b^	0.64 (0.57–0.71)	0.83 (0.74–0.93)	1.09 (0.96–1.22)	1.10 (0.97–1.24)	<0.001
IL-6 (pg/ml)^b^	1.22 (1.12–1.31)	1.34 (1.26–1.43)	1.51 (1.38–1.65)	1.57 (1.46–1.70)	<0.001
LBP (µg/ml)^b^	10.2 (8.9–11.7)	15.1 (13.0–17.5)	20.7 (17.8–24.1)	20.1 (17.2–23.5)	<0.001
HMW-Adiponectin (µg/ml)^b^	3.33 (2.99–3.72)	2.40 (2.14–2.69)	2.21 (1.93–2.53)	2.05 (1.82–2.31)	0.002

a Data are unadjusted arithmetic means (SD), *P* values were calculated after adjustment for age and sex.

b Data are unadjusted geometric means (95% CI).

c *P* value was calculated after adjustment for sex only.

d *P* value was calculated after adjustment for age only.

Percentages may not sum to 100 because of rounding.

### Association between IL-18 concentrations and body composition parameters

In the whole sample, plasma IL-18 was positively correlated with BMI, waist circumference, total body fat mass/percentage, total body lean mass, FMI and LMI after controlling for age and sex (all *P*<0.001, **Table 2**). Further controlling for FMI or LMI (mutually adjusted for each other) only abolished the significant correlations of IL-18 with FMI (*r* = 0.04, *P*>0.05), but not with LMI (*r* = 0.11, *P*<0.001).

**Table 2:** Partial spearman correlation coefficients between IL-18 and body composition, metabolic features and cytokines^a^.

	Total (n = 941)	Men (n = 345)	Women (n = 596)	Normal-weight(n = 448)	Overweight/obesity (n = 493)
BMI	0.19^e^	0.12^g^	0.21^e^	0.05	0.12^g^
Waist circumference	0.20^e^	0.12^g^	0.23^e^	0.08	0.13^f^
Total body fat %	0.13^e^	0.08	0.15^e^	0.01	0.06
Total body fat mass	0.16^e^	0.10	0.20^e^	0.03	0.10^g^
Total body lean mass	0.17^e^	0.13^g^	0.17^e^	0.01	0.12^g^
FMI	0.17^e^	0.10	0.20^e^	0.03	0.11^g^
FMI^b^	0.04	0.01	0.07	0.03	0.06
LMI	0.20^e^	0.14^g^	0.22^e^	0.05	0.17^e^
LMI^c^	0.11^e^	0.09	0.10^g^	0.05	0.14^f^
Systolic blood pressure^d^	0.09^f^	0.05	0.10^g^	0.06	0.11^g^
Diastolic blood pressure^d^	0.11^e^	0.06	0.12^f^	0.10^g^	0.10^g^
Total cholesterol^d^	−0.03	0.02	−0.05	−0.01	−0.05
HDL cholesterol^d^	−0.14^e^	−0.09	−0.16^e^	−0.13^f^	−0.15^f^
LDL cholesterol^d^	−0.01	0.01	−0.03	0.01	−0.03
Triglycerides^d^	0.09^f^	0.11^g^	0.09^g^	0.08	0.09^g^
Fasting glucose^d^	0.06	0.05	0.06	0.01	0.11^g^
HbA1c^d^	0.03	0.09	0.01	−0.05	0.11^g^
insulin^d^	0.11^e^	0.11^g^	0.11^f^	0.14^f^	0.08
HOMA-IR^d^	0.11^e^	0.12^g^	0.12^f^	0.14^f^	0.10^g^
CRP^d^	0.14^e^	0.15^f^	0.13^f^	0.12^g^	0.18^e^
IL-6^d^	0.11^f^	0.08	0.11^f^	0.14^f^	0.06
LBP^d^	0.15^e^	0.13^g^	0.15^e^	0.12^g^	0.19^e^
HMW-Adiponectin^d^	−0.09^f^	−0.01	−0.13^f^	−0.12^g^	−0.08

a All correlation coefficients were calculated after adjustment for age and sex, *P* value not adjusted for sex in sex-stratified models;

b Further adjusted for LMI;

c further adjusted for FMI;

d further adjusted for BMI.

e *P*<0.001,

f *P*<0.01,

g *P*<0.05.

IL-18 was also correlated with blood pressure, HDL cholesterol, triglycerides, insulin, HOMA-IR, inflammatory markers and HMW-adiponectin (all *P*<0.01, **Table 2**). The correlations of most metabolic traits were more pronounced in females and overweight/obese subjects than in their male and normal-weight counterparties.

### Association between IL-18 concentrations and metabolic syndrome

Overall, the ORs for MetS increased progressively across the IL-18 quartiles (*P*<0.001 for trend, **Table 3**) after adjusting for age, sex, lifestyle factors, family history of chronic diseases and BMI. The ORs for MetS comparing the highest with the lowest IL-18 quartile were 3.43 (95% confidence interval 2.01–5.85) (**model 2**). Additionally controlling for inflammatory markers (CRP, IL-6, LBP) (**model 3**) and HMW-adiponectin (**model 4**), the ORs of MetS and its components (elevated blood pressure and low HDL cholesterol) were attenuated slightly, but remained significant. Men and women showed similar trends for the IL-18-MetS association (**Table S1 and Table S2**) without a significant interaction between IL-18 and sex (*P* for interaction >0.05 for all models).

**Table 3:** Odds ratios and 95% confidence interval for metabolic syndrome according to quartile of IL-18^a^.

	Quartile of IL-18				
	Q1 (IL-18≤172.1 pg/ml)	Q2 (172.1<IL-18≤228.5 pg/ml)	Q3 (228.5<IL-18≤290.9 pg/ml)	Q4 (IL-18>290.9 pg/ml)	*P* for trend
**Metabolic syndrome**	**56/235**	**91/235**	**106/236**	**137/235**	
Model 1	1	1.92 (1.28–2.87)	2.38 (1.59–3.57)	4.00 (2.64–6.05)	<0.001
Model 2	1	1.63 (0.98–2.73)	1.54 (0.91–2.61)	3.43 (2.01–5.85)	<0.001
Model 3	1	1.64 (0.97–2.77)	1.36 (0.79–2.34)	3.18 (1.84–5.49)	<0.001
Model 4	1	1.56 (0.92–2.64)	1.36 (0.79–2.34)	3.10 (1.79–5.37)	<0.001
**Central obesity**	**97/235**	**122/235**	**129/236**	**137/235**	
Model 1	1	1.60 (1.11–2.32)	1.83 (1.25–2.66)	2.20 (1.49–3.24)	<0.001
Model 2	1	1.04 (0.52–2.12)	0.71 (0.34–1.49)	1.16 (0.55–2.46)	0.929
Model 3	1	1.05 (0.51–2.16)	0.65 (0.31–1.38)	1.09 (0.51–2.31)	0.887
Model 4	1	1.03 (0.50–2.11)	0.64 (0.30–1.36)	1.07 (0.50–2.27)	0.858
**Elevated blood pressure**	**59/235**	**90/235**	**104/236**	**134/235**	
Model 1	1	1.64 (1.09–2.46)	1.92 (1.28–2.88)	3.06 (2.03–4.63)	<0.001
Model 2	1	1.42 (0.92–2.21)	1.45 (0.93–2.28)	2.39 (1.52–3.75)	<0.001
Model 3	1	1.35 (0.87–2.12)	1.31 (0.83–2.06)	2.16 (1.37–3.40)	0.002
Model 4	1	1.35 (0.87–2.12)	1.31 (0.83–2.06)	2.16 (1.37–3.40)	0.002
**Hypertriglyceridemia**	**51/235**	**67/235**	**83/236**	**98/235**	
Model 1	1	1.19 (0.77–1.83)	1.47 (0.96–2.25)	1.74 (1.13–2.67)	0.007
Model 2	1	0.97 (0.62–1.54)	1.05 (0.66–1.66)	1.26 (0.80–1.99)	0.264
Model 3	1	0.90 (0.56–1.43)	0.88 (0.55–1.40)	1.08 (0.68–1.73)	0.688
Model 4	1	0.82 (0.51–1.31)	0.83 (0.52–1.34)	0.99 (0.62–1.60)	0.869
**Low HDL cholesterol**	**59/235**	**88/235**	**76/236**	**104/235**	
Model 1	1	1.98 (1.32–2.96)	1.66 (1.10–2.51)	2.95 (1.95–4.48)	<0.001
Model 2	1	1.71 (1.13–2.60)	1.24 (0.80–1.92)	2.36 (1.53–3.65)	0.001
Model 3	1	1.70 (1.11–2.58)	1.20 (0.77–1.87)	2.34 (1.51–3.63)	0.002
Model 4	1	1.55 (1.01–2.38)	1.14 (0.72–1.79)	2.16 (1.38–3.39)	0.005
**Hyperglycemia**	**133/235**	**155/235**	**160/236**	**160/235**	
Model 1	1	1.50 (1.03–2.20)	1.62 (1.10–2.39)	1.67 (1.12–2.49)	0.012
Model 2	1	1.39 (0.94–2.05)	1.43 (0.96–2.14)	1.43 (0.95–2.17)	0.098
Model 3	1	1.41 (0.95–2.09)	1.39 (0.92–2.10)	1.40 (0.92–2.14)	0.142
Model 4	1	1.41 (0.95–2.10)	1.39 (0.92–2.10)	1.40 (0.92–2.14)	0.144

a Metabolic syndrome and its components were defined based upon the updated National Cholesterol Education Program Adult Treatment Panel III criteria for Asian-Americans.

Model 1, adjusted for age and sex;

Model 2, further adjusted for smoking, alcohol drinking, physical activity, education, family history of chronic diseases and BMI;

Model 3, further adjusted for inflammatory markers (CRP, IL-6 and LBP);

Model 4, further adjusted for HMW-adiponectin.

**Figure 1:**
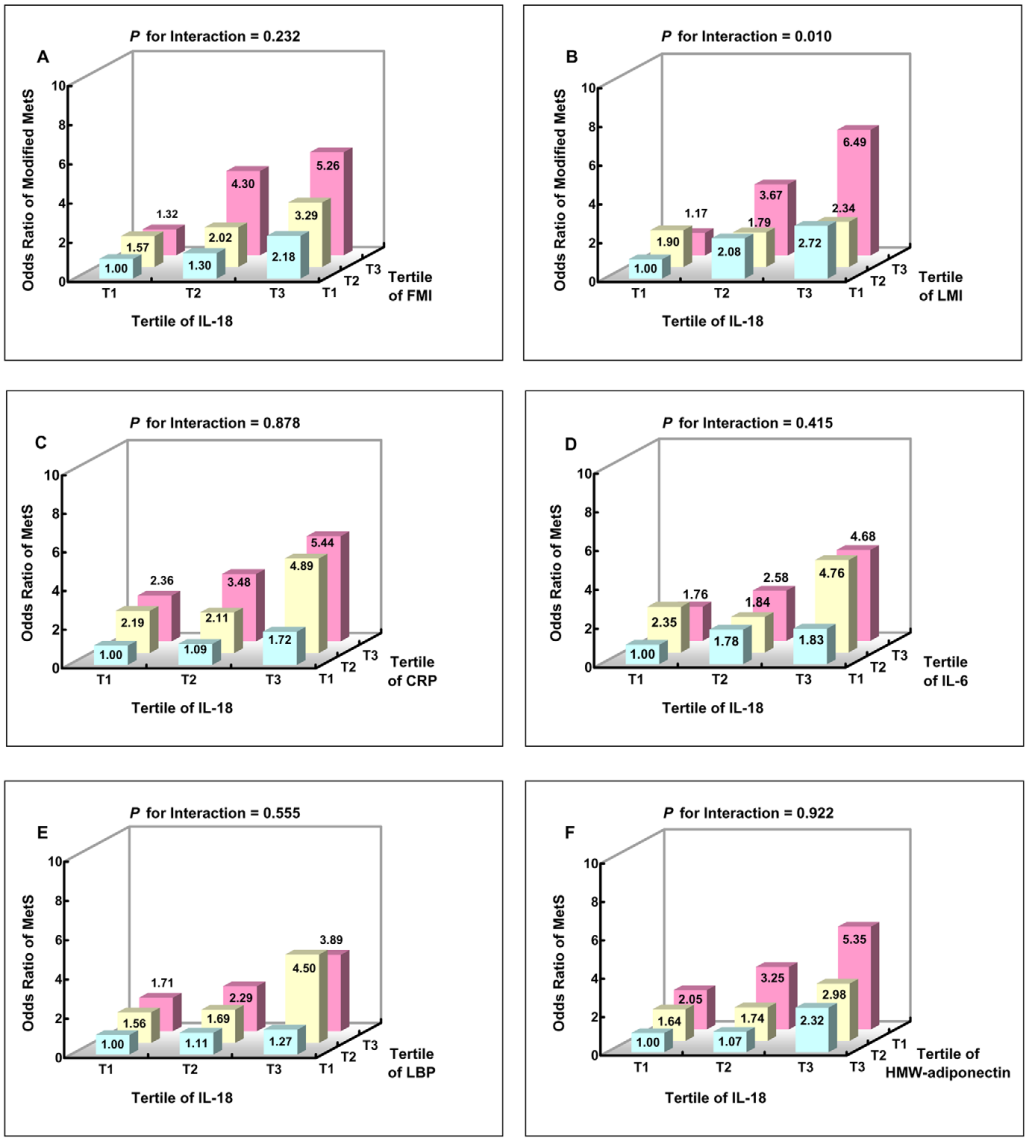
Odds ratio for metabolic syndrome according to joint classification of IL-18 and FMI (A), LMI (B) and cytokines (C–F). A to B: Modified metabolic syndrome was defined as having 2 or more components of metabolic syndrome without central obesity. Adjusted for age, sex, smoking, alcohol drinking, physical activity, education, family history of chronic diseases and LMI (A) or FMI (B). C to F: Adjusted for age, sex, smoking, alcohol drinking, physical activity, education, family histories of chronic diseases and BMI.

In the stratified multivariable regression analyses, the positive IL-18-MetS associations were independent of tertile of FMI, but significantly interacted with tertile of LMI (*P* for interaction = 0.232 and 0.010, respectively) (**Figure 1** **, A–B**). When further stratified by sex, significant interaction between IL-18 and LMI was only observed in women (*P* for interaction = 0.043) (**Figure S1**). Moreover, high IL-18 concentrations seemed to increase the ORs for MetS, regardless levels of CRP, IL-6, LBP and HMW-adiponectin (*P* for interaction = 0.878, 0.415, 0.555 and 0.922, respectively) (**Figure 1** **, C–F**).

## Discussion

We found a strong association between elevated plasma IL-18 and the MetS prevalence, independent of lifestyle factors, BMI, adipokine and inflammatory markers in apparently healthy Chinese. These results suggested that IL-18-MetS association might not be exclusively mediated by excess adiposity or associated biomarkers, and lean mass might also play a potential role. To our best knowledge, this is the first study to elucidate the effects of body compartments, distinguished by FMI and LMI, on IL-18 and its association with the MetS in Chinese.

Unlike the findings from most previous studies, we observed that LMI, but not FMI was possitively correlated with plasma IL-18 (**Table 2**), suggesting lean mass as a potential source of IL-18. Meanwhile, we also found significantly higher IL-18 levels in overweight/obese individuals than in normal-weight counterparts, consistent with the findings of others [18]. Excess adipose tissue was viewed as a major source of circulating IL-18 in many studies when BMI, containing the fractions of both fat and fat-free compartments [24], was used in analyses. Thus the effect of fat mass on IL-18 generation might be confounded by lean mass or vice versa. Besides adipocytes [3], non-adipocytes in adipose tissue could also secrete IL-18 [25], while TNF-α infusion in men increased *IL18* mRNA expression in muscles but not in adipose tissue [20]. Another plausible explanation for the discrepant results might be due to the fact that body composition was measured by bioelectrical impedance in some studies [10], [17] but by DXA in others [15], [16]. With more accurate measurement of DXA, particularly with large sample size like the Dallas Heart Study (n = 2231), both total of fat or lean mass was significantly correlated with circulating IL-18 [16]. However, the two compartments were not mutually adjusted for each other in that study, therefore it remains unclear to what degree the correlations were explained by fat or lean mass.

Our study provides evidence that elevated plasma IL-18 was a strong and independent risk factor of MetS (**Table 3**) and some of its components among Chinese, similar to the observation in a Caucasian population [11]. It was noteworthy that FMI, an indicator of adiposity, did not show much appreciable effect. On the contrary, the positive IL-18-MetS association was significantly interacted with tertile of LMI. In fact, lean mass or fat-free mass (combining both lean and bone masses) was previously suggested to be associated with MetS and its components. For example, obese women with MetS have more lean mass than those without MetS [26]. The results from a population-based study in Chinese showed that either fat or fat-free mass index was significantly and independently associated with MetS prevalence [23]. Moreover, metabolic inflexibility and insulin resistance in skeletal muscle are thought to be major contributors to metabolic disorders [27]. Skeletal muscle insulin resistance in young healthy subjects could promote atherogenic dyslipidemia at the early stage of MetS independent of intra-abdominal obesity and adipokines [28]. Obviously, whether mechanistic link exists between circulating IL-18 and skeletal muscle insulin resistance in pathogenesis of cardio-metabolic disorders needs to be elucidated further.

As a pro-inflammatory cytokine, IL-18 could stimulate the activation of NF-κB pathway and subsequently synthesize IL-6 and CRP [29]. However, even with significant correlation between these inflammatory markers and IL-18 levels in current study (**Table 2**), controlling for CRP and IL-6 did not substantially change the IL-18-MetS association, in agreement with previous studies [11], [12]. Thus, one possibility is that IL-18 might act through non-adiposity-related inflammatory pathways [13]. IL-18 is also a pleiotropic cytokine related with both innate and acquired immune responses [5] and one of its native stimuli is subclinical endotoxemia, indicated by low to moderately elevated circulating lipopolysaccharide (LPS) derived from gram-negative bacteria [7]. Recently, we found a positive association between elevated LBP and the MetS prevalence in this study population [21]. LBP plays an essential role in recognizing LPS and triggering the downstream inflammatory cascade. Therefore, it could be used as a biomarker reflecting innate immune response triggered by LPS [30]. However, the associations of MetS with LBP and IL-18 tended to be independent of each other in current analysis (**Figure 1E**). Thus, it appears that LPS-LBP induced innate immune response might not exert large impact on the association between IL-18 and the MetS prevalence.

Overall, the findings of our study have provided further insights into the sources of IL-18 as well as modifying factors, particularly the body-sized adjusted lean body mass, on the IL-18-MetS association. However, owing to the cross-sectional design, no causal relation could be established. The data were obtained from a case-control sample, which might limit the finding of our study to be generalized in general populations. Moreover, the measurement of body composition by DXA scan is unable to discriminate visceral adiposity and subcutaneous adiposity. Nonetheless, additional controlling for hepatic enzymes - markers of fatty liver, did not substantially change the results (data not shown). Furthermore, we could not rule out the possibility that the observed significant interaction between IL-18 and lean body mass on the MetS might be by chance because of multiple testing. Therefore, the findings for our study should be confirmed in prospective studies and also in different populations.

Our study suggests a strong positive association between circulating IL-18 levels and the MetS in apparently healthy Chinese, independent of obesity, fat mass index, adipokine and inflammation. More studies are needed to clarify the roles of lean mass in generating IL-18 and also its relationship with cardio-metabolic disorders.

## Supporting Information

Figure S1 **Odds ratio for modified metabolic syndrome according to joint classification of IL-18 and FMI or LMI in men (A and B) and women (C and D).** Adjusted for age, smoking, alcohol drinking, physical activity, education and family histories of chronic diseases and LMI (A and C) or FMI (B and D). Menopause status and hormone use were further adjusted for women.
(TIF)

Table S1 **Odds ratios and 95% confidence interval for metabolic syndrome according to tertile of IL-18 in men (n = 345).**
(DOC)

Table S2 **Odds ratios and 95% confidence interval for metabolic syndrome according to tertile of IL-18 in women (n = 596).**
(DOC)
