# Supplementary figures and images for: Lean Body Mass, Interleukin 18, and Metabolic Syndrome in Apparently Healthy Chinese
Source: PLoS One. 2011 Mar 18;6(3):e18104. doi: 10.1371/journal.pone.0018104 (PMC3060923; doi:10.1371/journal.pone.0018104)

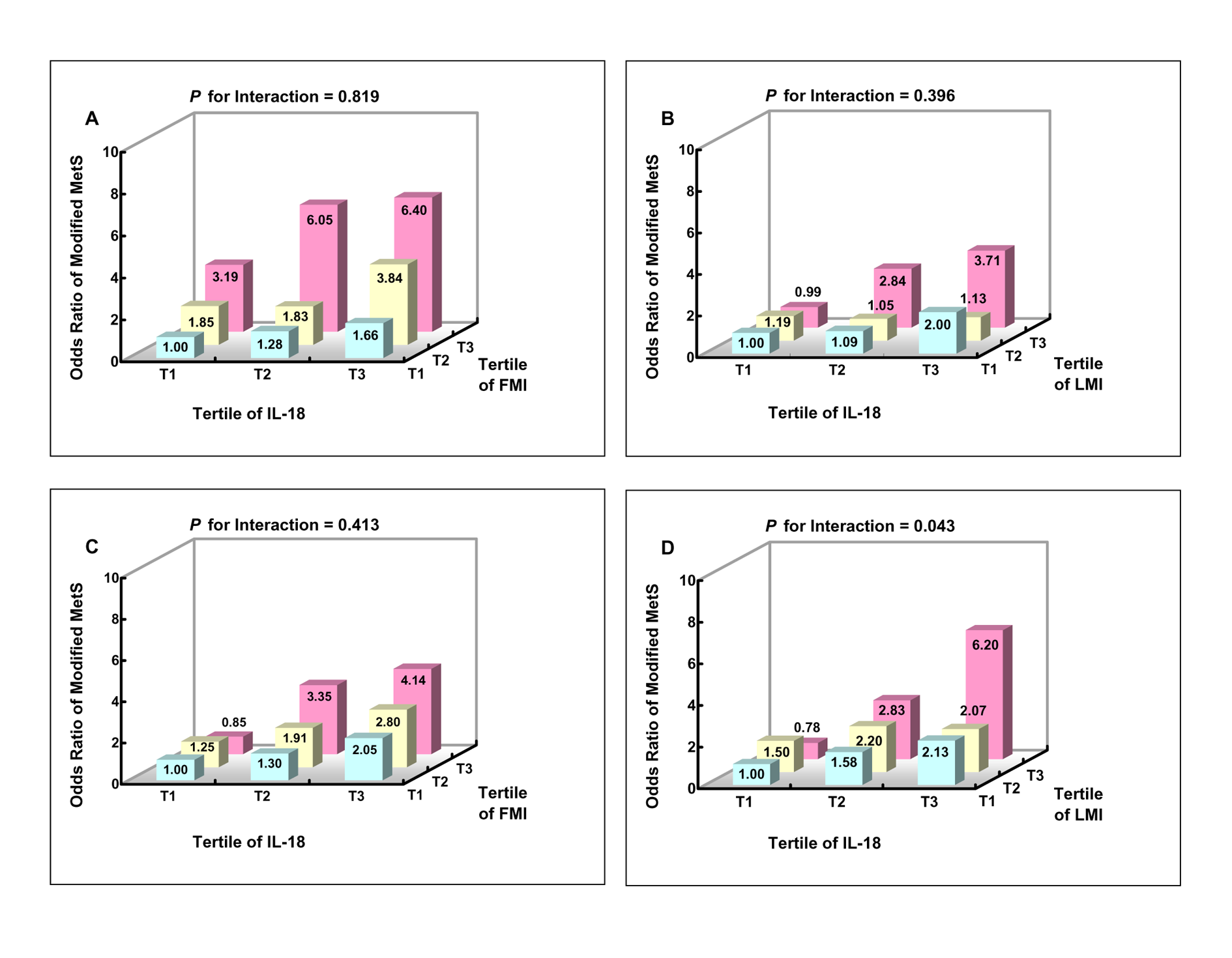

Supplement: Figure S1 — Odds ratio for modified metabolic syndrome according to joint classification of IL-18 and FMI or LMI in men (A and B) and women (C and D). Adjusted for age, smoking, alcohol drinking, physical activity, education and family histories of chronic diseases and LMI (A and C) or FMI (B and D). Menopause status and hormone use were further adjusted for women. (TIF) [file pone.0018104.s001.tif]
