# Supplementary material for: Lean Body Mass, Interleukin 18, and Metabolic Syndrome in Apparently Healthy Chinese
Source: PLoS One. 2011 Mar 18;6(3):e18104. doi: 10.1371/journal.pone.0018104 (PMC3060923; doi:10.1371/journal.pone.0018104)
Supplement: Table S1 — Odds ratios and 95% confidence interval for metabolic syndrome according to tertile of IL-18 in men (n = 345). (DOC) [file pone.0018104.s002.doc]

**Table S1 Odds ratios and 95% confidence interval for metabolic syndrome according to tertile of IL-18 in men (n=345).**

|  | **Tertile of IL-18** | | |  |
| --- | --- | --- | --- | --- |
|  | **T1 (IL-18≤228.4**  **pg/ml)** | **T2 (228.4<IL-18≤296.1pg/ml)** | **T3 (IL-18>296.1 pg/ml**) | ***P* for trend** |
| **Metabolic syndrome** | **44/115** | **56/115** | **69/115** |  |
| Model 1 | 1 | 1.53 (0.90-2.58) | 2.39 (1.40-4.06) | 0.001 |
| Model 2 | 1 | 1.24 (0.61-2.52) | 2.83 (1.38-5.79) | 0.004 |
| Model 3 | 1 | 1.12 (0.54-2.30) | 2.70 (1.30-5.58) | 0.007 |
| Model 4 | 1 | 1.13 (0.54-2.35) | 2.95 (1.41-6.20) | 0.004 |
| **Central obesity** | **52/115** | **58/115** | **62/115** |  |
| Model 1 | 1 | 1.23 (0.73-2.07) | 1.41 (0.84-2.37) | 0.194 |
| Model 2 | 1 | 0.52 (0.20-1.32) | 1.09 (0.42-2.82) | 0.844 |
| Model 3 | 1 | 0.45 (0.17-1.18) | 1.09 (0.41-2.86) | 0.838 |
| Model 4 | 1 | 0.45 (0.17-1.18) | 1.10 (0.42-2.89) | 0.823 |
| **Elevated blood pressure** | **52/115** | **64/115** | **68/115** |  |
| Model 1 | 1 | 1.52 (0.90-2.56) | 1.72 (1.02-2.91) | 0.043 |
| Model 2 | 1 | 1.36 (0.75-2.46) | 1.56 (0.87-2.81) | 0.139 |
| Model 3 | 1 | 1.28 (0.70-2.33) | 1.46 (0.80-2.65) | 0.216 |
| Model 4 | 1 | 1.28 (0.70-2.33) | 1.49 (0.82-2.72) | 0.193 |
| **Hypertriglyceridemia** | **48/115** | **54/115** | **58/115** |  |
| Model 1 | 1 | 1.24 (0.73-2.08) | 1.42 (0.84-2.39) | 0.187 |
| Model 2 | 1 | 1.04 (0.57-1.89) | 1.22 (0.68-2.19) | 0.509 |
| Model 3 | 1 | 0.95 (0.52-1.75) | 1.11 (0.61-2.02) | 0.719 |
| Model 4 | 1 | 0.93 (0.50-1.72) | 1.19 (0.65-2.19) | 0.569 |
| **Low HDL cholesterol** | **35/115** | **34/115** | **42/115** |  |
| Model 1 | 1 | 0.97 (0.55-1.71) | 1.36 (0.78-2.37) | 0.271 |
| Model 2 | 1 | 0.77 (0.42-1.41) | 1.19 (0.66-2.13) | 0.541 |
| Model 3 | 1 | 0.82 (0.44-1.52) | 1.17 (0.64-2.15) | 0.610 |
| Model 4 | 1 | 0.79 (0.42-1.48) | 1.29 (0.69-2.42) | 0.423 |
| **Hyperglycemia** | **70/115** | **77/115** | **74/115** |  |
| Model 1 | 1 | 1.30 (0.75-2.24) | 1.12 (0.65-1.93) | 0.674 |
| Model 2 | 1 | 1.25 (0.71-2.21) | 1.01 (0.58-1.77) | 0.966 |
| Model 3 | 1 | 1.25 (0.70-2.24) | 0.92 (0.52-1.64) | 0.787 |
| Model 4 | 1 | 1.25 (0.70-2.24) | 0.93 (0.52-1.66) | 0.820 |

Model 1, adjusted for age;

Model 2, further adjusted for smoking, alcohol drinking, physical activity, education, family histories of chronic diseases and BMI;

Model 3, further adjusted for inflammatory markers (CRP, IL-6 and LBP);

Model 4, further adjusted for HMW-adiponectin.
