# Supplementary material for: Lean Body Mass, Interleukin 18, and Metabolic Syndrome in Apparently Healthy Chinese
Source: PLoS One. 2011 Mar 18;6(3):e18104. doi: 10.1371/journal.pone.0018104 (PMC3060923; doi:10.1371/journal.pone.0018104)
Supplement: Table S2 — Odds ratios and 95% confidence interval for metabolic syndrome according to tertile of IL-18 in women (n = 596). (DOC) [file pone.0018104.s003.doc]

**Table S2 Odds ratios and 95% confidence interval for metabolic syndrome according to tertile of IL-18 in women (n=596).**

|  | **Tertile of IL-18** | | |  |
| --- | --- | --- | --- | --- |
|  | **T1 (IL-18≤171.5 pg/ml)** | **T2 (171.5<IL-18≤245.2pg/ml)** | **T3 (IL-18>245.2 pg/ml**) | ***P* for trend** |
| **Metabolic syndrome** | **45/199** | **73/198** | **103/199** |  |
| Model 1 | 1 | 1.92 (1.23-2.99) | 3.51 (2.27-5.44) | <0.001 |
| Model 2 | 1 | 1.71 (0.97-3.00) | 2.27 (1.30-3.97) | 0.004 |
| Model 3 | 1 | 1.64 (0.92-2.93) | 2.06 (1.16-3.66) | 0.015 |
| Model 4 | 1 | 1.60 (0.89-2.88) | 1.95 (1.09-3.49) | 0.026 |
| **Central obesity** | **84/199** | **101/198** | **128/199** |  |
| Model 1 | 1 | 1.39 (0.93-2.07) | 2.40 (1.60-3.59) | <0.001 |
| Model 2 | 1 | 0.68 (0.30-1.53) | 1.08 (0.47-2.49) | 0.905 |
| Model 3 | 1 | 0.66 (0.29-1.52) | 0.99 (0.43-2.31) | 0.932 |
| Model 4 | 1 | 0.64 (0.28-1.49) | 0.96 (0.41-2.24) | 0.872 |
| **Elevated blood pressure** | **45/199** | **67/198** | **91/199** |  |
| Model 1 | 1 | 1.63 (1.04-2.57) | 2.70 (1.73-4.21) | <0.001 |
| Model 2 | 1 | 1.60 (0.97-2.63) | 2.10 (1.28-3.45) | 0.003 |
| Model 3 | 1 | 1.46 (0.88-2.43) | 1.86 (1.13-3.08) | 0.016 |
| Model 4 | 1 | 1.47 (0.89-2.45) | 1.89 (1.14-3.14) | 0.014 |
| **Hypertriglyceridemia** | **37/199** | **43/198** | **59/199** |  |
| Model 1 | 1 | 1.13 (0.69-1.86) | 1.71 (1.06-2.75) | 0.025 |
| Model 2 | 1 | 0.97 (0.57-1.63) | 1.23 (0.74-2.05) | 0.389 |
| Model 3 | 1 | 0.84 (0.49-1.44) | 1.03 (0.61-1.74) | 0.846 |
| Model 4 | 1 | 0.79 (0.45-1.36) | 0.92 (0.54-1.56) | 0.812 |
| **Low HDL cholesterol** | **53/199** | **69/198** | **94/199** |  |
| Model 1 | 1 | 1.53 (0.99-2.36) | 2.60 (1.70-3.97) | <0.001 |
| Model 2 | 1 | 1.35 (0.85-2.14) | 1.99 (1.26-3.15) | 0.003 |
| Model 3 | 1 | 1.35 (0.85-2.14) | 2.01 (1.26-3.21) | 0.003 |
| Model 4 | 1 | 1.28 (0.79-2.06) | 1.80 (1.12-2.91) | 0.015 |
| **Hyperglycemia** | **113/199** | **135/198** | **139/199** |  |
| Model 1 | 1 | 1.57 (1.04-2.37) | 1.68 (1.11-2.55) | 0.014 |
| Model 2 | 1 | 1.46 (0.95-2.23) | 1.36 (0.88-2.10) | 0.161 |
| Model 3 | 1 | 1.51 (0.98-2.32) | 1.38 (0.88-2.16) | 0.148 |
| Model 4 | 1 | 1.51 (0.98-2.33) | 1.39 (0.88-2.18) | 0.144 |

Model 1, adjusted for age;

Model 2, further adjusted for smoking, alcohol drinking, physical activity, education, family histories of chronic diseases, menopause status, hormone use and BMI;

Model 3, further adjusted for inflammatory markers (CRP, IL-6 and LBP);

Model 4, further adjusted for HMW-adiponectin.
